# Relegating malaria resurgences to history

**DOI:** 10.1186/1475-2875-11-123

**Published:** 2012-04-24

**Authors:** Robert D Newman

**Affiliations:** 1 Global Malaria Programme, World Health Organization, 20, Avenue Appia, CH-1211, Geneva, 27, Switzerland

**Keywords:** Malaria, Resurgence, Financing

## Abstract

Progress in malaria control over the past decade has been striking, with malaria mortality rates falling by approximately one quarter globally and more than a third in the World Health Organization African Region. In the accompanying paper, Cohen *et al.* demonstrate the potential fragility of these gains, comprehensively describing malaria resurgences that have occurred over the past 80 or so years. They found that the vast majority of resurgences were due, at least in part, to the weakening of malaria control programmes; resource constraints were the most commonly identified factor. Their findings are timely and compelling, demonstrating that global efforts will be wasted if the required resources are not secured to achieve *and* maintain universal access to life-saving malaria prevention and control tools. The greatest threats to current malaria control efforts are not biological, but financial. The increases in funding for malaria over the past decade, while impressive, still fall far short of the nearly $6 billion dollars required annually. Domestic spending by endemic country governments on malaria specifically, and health more generally, could go a long way towards filling the projected funding gap. However, external funding is also essential, and the global community needs to work together to ensure full funding of the Global Fund to Fight AIDS, Tuberculosis, and Malaria, which has been the single largest source of malaria funding over the past decade. This year, on April 25th, World Malaria Day will be celebrated with the theme Sustain Gains, Save Lives: Invest in Malaria. The review by Cohen *et al.* suggests one possible future if such investment is not made. However, with sufficient support, malaria resurgences can be relegated to history.

## 

Over the past decade, malaria has been reinstated as a priority for global health investment. International funding commitments for malaria control have risen from under $100 million in 2003 to more than $2 billion in 2011[1]. These resources have allowed for the scale-up of proven malaria control interventions, including long-lasting insecticidal nets (LLINs), indoor residual spraying, diagnostic testing, and treatment with highly efficacious artemisinin-based combination therapy. As an example, more than 290 million LLINs were distributed in sub-Saharan Africa between 2008 and the end of 2010 [1].

Concurrent with the dramatic increases in access to malaria control interventions, malaria cases have fallen by more than 50% in 43 countries (eight of them in Africa) over the past decade; another eight countries have registered declines of more than 25% [1]. While many of these declines occurred in countries with already low malaria transmission, some have occurred in settings with moderate-to-high transmission. Over this same time period, malaria mortality rates have fallen by approximately one quarter globally and more than a third in the WHO African Region [1].

While the progress in malaria control has been striking, it is frequently described as fragile. In the accompanying paper, Cohen *et al.* demonstrate just how true this is, comprehensively describing malaria resurgences that have occurred over the past 80 or so years [2]. They found that the vast majority (more than 90%) of resurgences were due, at least in part, to the weakening of malaria control programmes; resource constraints were the most commonly identified factor. Less than one third of resurgences were associated with drug or insecticide resistance.

Their findings are timely and compelling. While addressing *Plasmodium* resistance to anti-malarial medicines and *Anopheles* resistance to insecticides are urgent priorities requiring concerted global action [3,4], such efforts will be wasted if the required resources are not secured to achieve *and* maintain universal access to life-saving malaria prevention and control tools. The greatest threats to current malaria control efforts are not biological, but financial.

The increases in funding for malaria over the past decade, while impressive, still fall far short of the nearly $6 billion dollars required annually to ensure universal access to malaria prevention and control [5]. Despite stable and significant funding from the United States through the Presidents Malaria Initiative, and recent substantial commitments from the UK through the Department for International Development, the financial projections for the coming years are concerning. Based on current information, resources available for global malaria efforts are projected to remain relatively stable in 20122013, and then decline through 2015 [1].

That downward trend can, however, be reversed. First, increased domestic spending by endemic country governments on malaria specifically, and health more generally, could go a long way towards filling the projected funding gap. If just 1% of total domestic spending were made available for malaria control, 75 of the 99 countries with ongoing malaria transmission could raise enough to provide each person at risk with access to an ITN [1]. Second, the global community needs to work together to ensure full funding of the Global Fund to Fight AIDS, Tuberculosis, and Malaria. The Global Fund has been the single largest source of malaria funding over the past decade. The failure to fully replenish the Fund places a decade of progress at major risk. The cancellation of Round 11, and the limited pool of funding available through the Transitional Funding Mechanism, risk causing the very sort of service interruptions that Cohen *at al* have identified as being a primary factor in documented malaria resurgences.

In the case of malaria, the fact that the Global Fund has not categorized malaria prevention and control interventions as being eligible for Continuity of Services funding, as is the case for anti-retroviral and anti-tuberculosis medicines, medicines to prevent mother to child transmission of HIV, and opioid substitution therapy, only heightens the risk of reversing the gains in malaria control following service interruptions [6]. The failure to replace an LLIN before it is worn out places individual lives at risk, especially as continuous protection against malaria diminishes the acquisition of partial immunity. The failure to replace a cohort of LLINs in a timely manner places entire populations at risk of dramatic resurgences in malaria transmission, as demonstrated by Cohen *et al.*[2].

But such population-level reversals are not inevitable. What Cohen *et al.*[2] discuss only briefly are the countries that have successfully controlled or eliminated malaria and then maintained those achievements. Malaysia, Thailand, and Viet Nam, for example, have substantially reduced malaria cases and deaths since the early 1990s by intensifying and maintaining preventive efforts and ensuring wide availability of anti-malarial medicines. Reunion and Singapore have been able to maintain their malaria free status despite a continual risk of importation [1]. This sort of durable progress is an integral part not only of reaching ambitious global malaria targets, but of reaching the health-related Millennium Development Goals by 2015.

These successes demonstrate that the 43 countries where malaria cases have fallen by more than half over the past decade are not doomed to resurgence: malaria control *does* work, and the gains *can* be maintained. Such progress need not be ephemeral. Accomplishing this, however, requires continued political and financial commitment not only on the part of donors, but by endemic countries themselves.

This year, on April 25th, World Malaria Day will be celebrated with the theme Sustain Gains, Save Lives: Invest in Malaria. The review by Cohen *et al.*[2] suggests one possible future if such investment is not made. However, with sufficient support, malaria resurgences can be relegated to history.

## Competing interest

The author declares that he has no competing interests.

## Disclaimer

The views expressed in this publication are those of the author and do not necessarily represent the decisions, policy, or views of the World Health Organization.
